# The impact of ultra-processed foods and unprocessed or minimally processed foods on the quality of life among adolescents: a longitudinal study from China

**DOI:** 10.3389/fnut.2024.1489067

**Published:** 2024-11-20

**Authors:** Yuke Yang, Yan Gao, Xiangren Yi, Yining Hu, Liangyu Zhao, Lu Chen, Wenze Sui, Shuoqin Zhang, Sen Ma

**Affiliations:** School of Physical Education, Shandong University, Jinan, China

**Keywords:** ultra-processed foods, unprocessed or minimally processed foods, quality of life, Chinese adolescents, fixed effects model, gender difference

## Abstract

**Introduction:**

The purpose of this study was to explore the associations between ultra-processed foods (UPF), unprocessed or minimally processed foods (UMFs) with the quality of life (QoL) in Chinese adolescents.

**Methods:**

The study included a baseline survey in 2021 (T1) and a follow-up survey in 2022 (T2), with a total of 3,206 participants, including 1,510 males (Age ± SE: 13.62 ± 1.69) and 1,696 females (Age ± SE: 14.09 ± 1.85). QoL was measured using the Quality of Life Scale for Children and Adolescents. All adolescents were asked to recall the foods they had eaten in the past week, which was classified using the NOVA system.

**Results:**

Instant noodles, sugary drinks, and fried foods had a negative impact on adolescents’ QoL. Snacks or desserts only had a negative impact on the girls’ QoL. However, processed meats had a positive impact on the QoL of all adolescents. Fast food was beneficial for girls’ QoL. Fruits had a positive impact on the QoL of all adolescents. Green leafy vegetables, red and orange vegetables, fish, shrimp, or other seafood had a positive impact on the QoL of girls. Fresh poultry, pork, beef, and other meats had a positive impact on boys’ QoL. Soy products were detrimental to girls’ QoL. Tubers had a negative impact on boys’ QoL. Potatoes had a negative effect on the all adolescents’ QoL.

**Discussion:**

This study further expands the understanding of the relationship between UPF, UMFs, and QoL. It provides new insights and evidence for improving the QoL of adolescents.

## 1 Introduction

Quality of life (QoL), as conceptualized by the World Health Organization (WHO), is defined as “the individual’s perception of their status in life, within the context of the culture and value systems in which they live, and in relation to their goals, expectations, standards, and concerns” (1). Adolescence represents an exceedingly unique phase in life, characterized by rapid physical development, the formation of personality, and the refinement of character (2). Assessing the QoL of adolescents not only provides a comprehensive understanding of their happiness but also yields insights into their physiological functions, psychological states, and social interactions (3, 4).

Among adolescents, developmental delays and excessive weight are disturbingly frequent yet unresolved significant issues. It is estimated that globally, around 162 million children under the age of five are grappling with stunting (5), an issue closely linked to increased risks of early death and impaired development (6). The obesity crisis is equally concerning, with the projected prevalence of overweight among children under five set to climb from 7% in 2012 to 11% by 2025 (7). Obesity has emerged as a deeply ingrained risk factor affecting both the physical and mental well-being of adolescents, transcending geographic boundaries and societal strata (8). In light of these realities, it is an imperative matter of focus to attending to the nutritional and psychosocial development of school-aged children and adolescents, in order to foster a healthy progression from adolescence to adulthood (9).

Nutrition plays an integral role in the QoL for all demographics (10–12). Studies have shown that a diet of high quality (13), with increased consumption of dietary fiber (14), higher protein intake (15), and abundant fruits and vegetables with minimal sugar (16) can significantly promote physical and mental well-being and enhance QoL. However, research conducted by scholars suggests that China’s shift from a traditional dietary pattern to a Western one may pose potential health risks (17). On one hand, there has been a noticeable increase in the consumption of ultra-processed foods (UPF), such as snacks, sugary beverages, fast food, and cakes, which are high in sugar and fat (18, 19); on the other hand, the consumption of unprocessed or minimally processed foods (UMFs), including grains, vegetables, and fruits, is less than satisfactory (17).

Moreover, we found that nutrition is closely related to QoL. A recent study reported that the increase in UPF is associated with a decline in the QoL (11), especially among adolescents (20). In contrast to UPF, there is a growing body of evidence that UMFs can enhance QoL (12). Additionally, consumption of UMFs such as vegetables, beef, and milk by children has been shown to have a beneficial effect on their high-density lipoprotein cholesterol (HDL-C) levels (21). However, the effects of different types of UPF and UMFs intake on QoL have not been systematically studied among Chinese adolescents. Therefore, understanding the intake frequencies of different types of UPF and UMFs and their impact on the QoL of adolescents is of paramount importance.

To sum up, the aim of this study is to investigate the impact of UMFs and UPF on QoL in adolescents. On the basis of previous studies, we discussed in detail the effects of different types of UPF and UMFs on QoL, and analyzed the gender differences. It provides insight and new thinking on nutrition that can be used to intervene in the QoL of adolescents.

## 2 Materials and methods

### 2.1 Participants

This study is a survey research. We conducted a longitudinal survey according to the guidelines in the Helsinki Declaration. All procedures involving human subjects were approved by the Ethics Committee of Shandong University (20180517). Prior to the survey, parents and students both completed informed consent forms. Utilizing the population proportionate sampling (PPS) method, based on geographical, demographic, and socio-economic level, participants were randomly selected from 186 middle and high schools in 17 cities in Shandong Province from 2021 to 2022 (22, 23). One year later, we conducted a follow-up survey to obtain data from 2022 to 2023. All surveys are completed annually. Data collection involved on-site testing, questionnaires. During the collection process, trained surveyors used standardized guidelines to organize students to measure physical fitness and guide students in answering online questionnaires. All data were voluntary, anonymous, and confidential. The collected data were stored on a password-protected website (Database of Youth Health): https://www.ncmi.cn//phda/dataDetails.do?id=CSTR:17970.11.A0031.202107.209.V1.0

A total of 17,084 samples were collected for 2021–2022, and 16,494 for 2022–2023. Firstly, we tracked and matched the collected samples in two waves of data. Because of the inclusion of students in the graduation year of junior and senior high school at baseline, our survey conducted during the follow-up period was no longer able to track these graduates. Secondly, we excluded missing data for age, gender, SES vacancies. Finally, we also excluded UPF, UMFs, and QoL vacancies. After this, a total of 3,206 effective samples were obtained. The sample included 1,510 males (average age 13.62 ± 1.69) and 1,696 females (average age 14.09 ± 1.85).

### 2.2 Measures

#### 2.2.1 According to the NOVA food classification system

All adolescents were asked to recall and fill out the types and frequencies of foods they consumed in the past week. The NOVA system categorizes foods into four types based on the extent and purpose of industrial processing (24), which are: (1) UMFs: These include fresh fruits, vegetables, milk, legumes, and other foods that are essentially free of additives or excessive processing steps. All processes in these foods are aimed at making them edible. (2) Processed culinary ingredients: These are ingredients extracted from foods, such as salt, sugar, vegetable oil, or butter, which are used for cooking, boiling, and seasoning. (3) Processed foods: These are foods made by combining ingredients from the second category with those from the first category, such as canned vegetables or canned fruits. (4) UPF: These are the most processed foods, typically containing industrial food additives such as colorants, flavor enhancers, sweeteners, and emulsifiers. This system has been extensively utilized in a wide array of studies (25).

In this study, based on NOVA, variables were categorized and extracted (26), while also considering the local dietary habits and methods in China. Specifically, UPF were divided into six categories: processed meats (such as sausages, frankfurters, and salami), instant noodles, fast food (including items from McDonald’s, KFC, Pizza Hut, or other fast-food restaurants, such as hamburgers, fried chicken, fried fish fillets, French fries, and pizzas), sweet or salty snacks and desserts (such as cakes, cookies, candies, potato chips, or shrimp sticks), sugary beverages (such as soda, sugary milk drinks, or sugary fruit juice drinks), and fried foods.

Following the dietary habits in China, the following foods were included in the UMFs category: green leafy vegetables; red and orange vegetables (such as carrots and tomatoes); potatoes (excluding fried potatoes, fried potato chips, or potato chips); tubers (such as sweet potatoes, yams, and taro); fruits; soy products (such as tofu and other soy-based products); fresh poultry, pork, beef, or other meats; fish, shrimp, or other seafood; eggs; dairy products.

Food intake frequency was categorized into five levels: 0 times a week, 1–2 times a week, 3–5 times a week (once every other day), 6–7 times a week (once a day), or 8 times or more a week (more than once a day), corresponding to scores of 1–5. The total score for UPF and UMFs is calculated by adding the scores for all food categories. Among them, the UPF total score ranges from 6 to 30. The UMFs total score ranges from 10 to 50.

#### 2.2.2 Quality of life

QoL of children and adolescents was assessed using the Quality of Life Scale for Children and Adolescents (QLSCA). This questionnaire includes factors related to social and psychological functioning, physical and mental health, and living environment. It covers dimensions such as teacher-student relationships, peer relationships, parent–child relationships, learning abilities and attitudes, self-concept, physical sensation, negative emotions, work attitudes, convenience in life, opportunities for activities, and physical fitness. There are 49 questions, each with four response options: never, rarely, often, and always, corresponding to scores of 1–4. The scoring range is 49–196, with higher scores indicating better QoL. In order to test the reliability and validity of the QLSCA, exploratory factor analysis was conducted to examine the construct validity of the scale (KMO = 0.976, *p* = 0.000), and Cronbach’s alpha was used to test its reliability (Cronbach’s alpha = 0.950). The results indicated good reliability and validity. This scale has been widely used among Chinese adolescents (27).

#### 2.2.3 Covariates

In this study, we also selected control variables that might affect the results, including whether the child is an only child (1 = yes; 2 = no), whether they live in a dormitory (1 = yes; 2 = no), and socioeconomic status (SES). Among them, only child refers to a child who has no siblings and whose parents have raised only one child. Non-only children means that a child with siblings has two or more children in their family (28). These variables have been proven to affect the diet and health of adolescents by scholars (29). Among them, SES is closely related to the diet of adolescents, so we included it as a control variable (30). SES includes the education level of the father and mother (with 9 options, ranging from uneducated (It means no education, even in primary school) = 1, primary school = 2, junior high school = 3, technical school = 4, vocational high school = 5, high school = 6, college diploma = 7, bachelor’s degree = 8, graduate or above = 9), parents’ occupations (with 12 options, including unemployed, laid-off, farmer, self-employed, business and service workers, general workers, skilled workers, private entrepreneurs, ordinary clerks, technical workers, teachers, engineers, doctors, lawyers, or other professionals, middle or senior managers in enterprises or companies, leaders or department heads in government institutions or public institutions), and self-rated family economic conditions, consisting of five questions.

### 2.3 Data analysis

The descriptive analysis in this paper is based on baseline data (2021–2022). We used chi-square analysis and independent samples t-tests to test the gender differences in UPF, UMFs, and QoL. After that, using 2 years of data (Baseline: 2021–2022; Follow-up: 2022–2023), fixed effects (FE) were used to validate the relationship between the explanatory variables – UPF and UMFs, and the dependent variable – QoL. We also used the Hausman test to determine whether to use FE or random effects (RE). The Hausman test result indicated that the null hypothesis was rejected, suggesting that the FE was more suitable for this study. Therefore, we adopted the FE to test the relationship between food and QoL. In the analysis process, descriptive analysis was conducted using SPSS 27.0, and Hausman tests, robustness, heterogeneity, Variance Inflation Factor (VIF) and FE were performed using Stata 17.0.

To account for unobserved heterogeneity at the individual level, we conducted longitudinal estimates and heterogeneity analysis using data from two waves. Furthermore, to test the robustness of this study, the robustness of the t-statistics was verified to enhance robustness. To test for heterogeneity, differences were analyzed for gender and different types of UPF. To test for multicollinearity among variables, VIF calculations were conducted. The FE used in this study mitigated potential endogeneity issues.

## 3 Results

### 3.1 Descriptive analysis

Table 1 displays the characteristics of male and female participants in the baseline data (2021–2022). Chi-square analysis and *t*-tests showed that among the 3,206 participants, there were 1,510 males with an average age of 13.62 ± 1.69 years and 1,696 females with an average age of 14.09 ± 1.85 years. There were 1,339 only children (Age ± SE = 14.26 ± 2.02) and 1,867 (Age ± SE = 14.45 ± 1.73) non-only children. Approximately 35% of the students had parents with a junior high school education level, followed by high school. Additionally, we found significant gender differences in age, being an only child, parents’ education level, SES, and QoL: (1) Compared to female adolescents, a higher proportion of male adolescents were only children (44.11% vs. 39.68%), and a greater proportion of girls were non-only children (55.89% vs. 60.32%). (2) Boys were more likely to have fathers with no education, primary school, junior high school, and bachelor’s degrees than girls. Conversely, girls had fathers with a higher likelihood of technical school, vocational high school, high school, college, and postgraduate degrees than boys. (3) Boys were more likely to have mothers with no education, junior high school, vocational high school, bachelor’s, and postgraduate degrees than girls, while girls had mothers with a higher likelihood of primary school, technical school, high school, and college degrees than boys. (4) Girls had a higher family SES than boys. (5) Boys had a higher QoL than girls.

**Table 1:** Sociodemographic information and gender differences of Chinese adolescents at baseline (*N* = 3,206).

Characteristics	Sex *N* (%) / Mean ± SE		χ2 /t	*p*
	Boys	Girls		
**Only child status**			6.429	0.011*
Only child	666 (44.11)	673 (39.68)		
Non only children	844 (55.89)	1,023 (60.32)		
**Father’s education**			22.979	0.003**
Uneducated	34 (2.25)	23 (1.36)		
Primary school	102 (6.75)	105 (6.19)		
Junior high school	556 (36.82)	551 (32.49)		
Technical school	168 (11.13)	209 (12.32)		
Vocational high school	83 (5.50)	97 (5.72)		
Senior high school	240 (15.89)	320 (18.87)		
Junior college	145 (9.60)	214 (12.62)		
Bachelor degree	132 (8.74)	119 (7.02)		
Master degree or above	50 (3.31)	58 (3.42)		
**Mother’s education**			69.138	0.000**
Uneducated	44 (2.91)	29 (1.71)		
Primary school	148 (9.80)	189 (11.14)		
Junior high school	578 (38.28)	557 (32.84)		
Technical school	143 (9.47)	201 (11.85)		
Vocational high school	72 (4.77)	79 (4.66)		
Senior high school	213 (14.11)	275 (16.21)		
Junior college	111 (7.35)	225 (13.27)		
Bachelor degree	143 (9.47)	114 (6.72)		
Master degree or above	58 (3.84)	27 (1.59)		
**Age**	13.62 ± 1.69	14.09 ± 1.85	−7.641	0.000**
**SES**	22.22 ± 8.87	22.86 ± 8.37	−2.088	0.037*
**QoL**	147.11 ± 24.17	143.16 ± 23.06	4.713	0.000**
Total	1,510	1,696		

* *p* < 0.05, ** *p* < 0.01; SES, socioeconomic status; QoL, quality of life.

### 3.2 Ultra-processed foods

**Figure 1:**
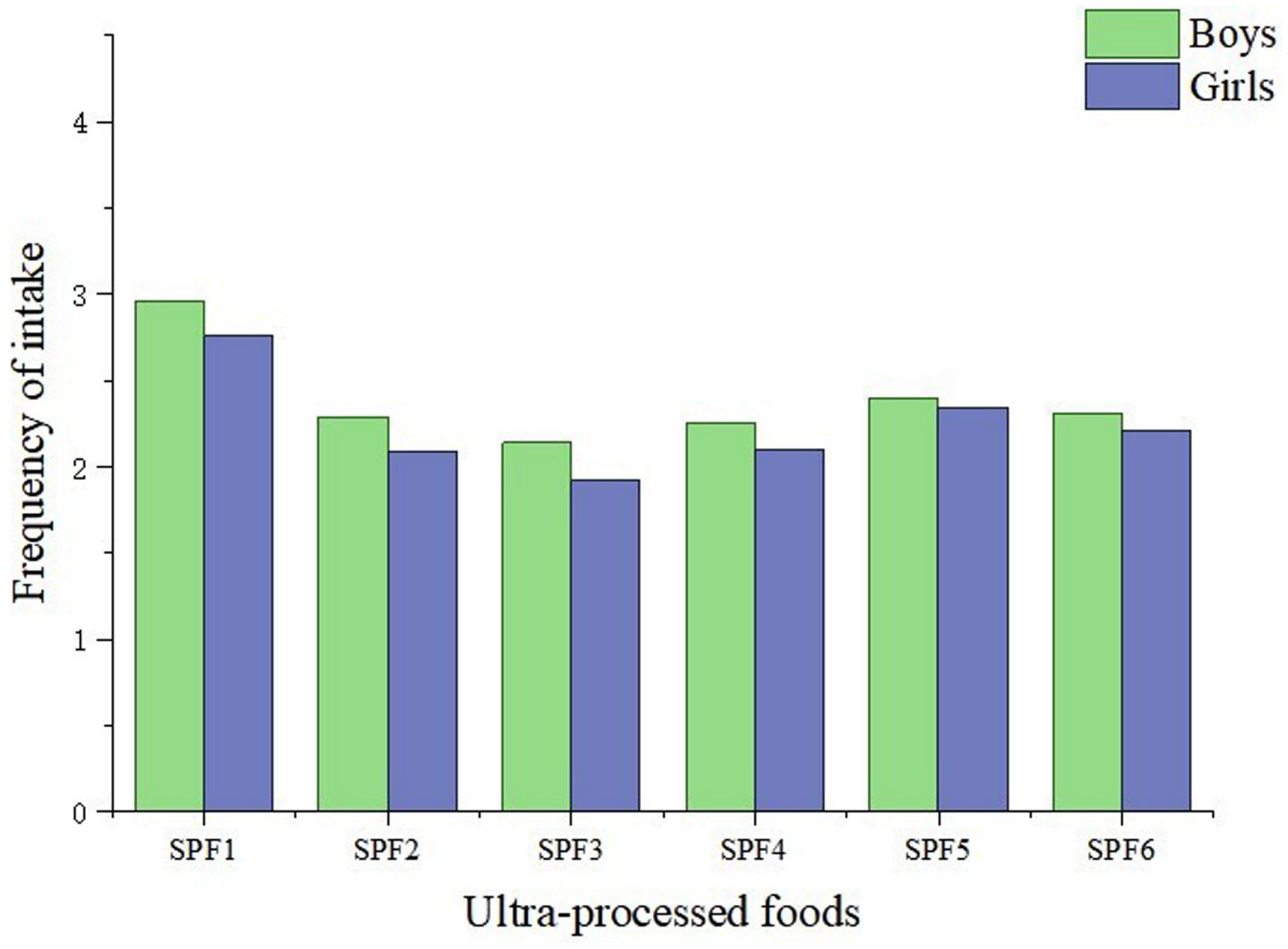
Gender differences in ultra-processed foods intake in two waves of data (*N* = 3,206). UPF1, processed meats; UPF2, instant noodles; UPF3, western fast food; UPF4, sugary beverages; UPF5, snacks and desserts; UPF6, fried foods.

Table 2 shows the consumption frequency of UPF among all students, with the first wave data (2021–2022) used as an example. The most popular UPF among adolescents was processed meats (sausages, etc.), with over 55% of adolescents consuming them at least 3–5 times per week. Following this were snacks or desserts, with about 35% of adolescents consuming them at least 3–5 times per week. Consumption frequency of fast food (food purchased from fast-food restaurants like McDonald’s, KFC, Pizza Hut, etc.) was the lowest, with about 40% of adolescents consuming it 0 times per week, and 40% consuming it 1–2 times per week. Additionally, there were differences in UPF intake between boys and girls. Boys were significantly more likely to consume UPF more than 8 times per week than girls, indicating that boys were more likely to consume more UPF weekly than girls. A visualization of gender differences is shown in Figure 1.

**Table 2:** Intake of ultra-processed foods and gender differences of Chinese adolescents at follow-up (*N* = 3,206).

Item	Characteristics	Sex (%)		Total	χ2	*p*
		Boys	Girls			
Processed meats	0 times per week	147 (9.74)	166 (9.79)	313 (9.76)	52.339	0.000**
	1–2 times per week	441 (29.21)	667 (39.33)	1,108 (34.56)		
	3–5 times per week	447 (29.60)	492 (29.01)	939 (29.29)		
	6–7 times per week	292 (19.34)	217 (12.79)	509 (15.88)		
	≥8 times per week	183 (12.12)	154 (9.08)	337 (10.51)		
Instant noodles	0 times per week	385 (25.50)	627 (36.97)	1,012 (31.57)	71.507	0.000**
	1–2 times per week	677 (44.83)	689 (40.63)	1,366 (42.61)		
	3–5 times per week	211 (13.97)	208 (12.26)	419 (13.07)		
	6–7 times per week	126 (8.34)	123 (7.25)	249 (7.77)		
	≥8 times per week	111 (7.35)	49 (2.89)	160 (4.99)		
Western fast food	0 times per week	551 (36.49)	740 (43.63)	1,291 (40.27)	61.231	0.000**
	1–2 times per week	571 (37.81)	706 (41.63)	1,277 (39.83)		
	3–5 times per week	186 (12.32)	121 (7.13)	307 (9.58)		
	6–7 times per week	102 (6.75)	66 (3.89)	168 (5.24)		
	≥8 times per week	100 (6.62)	63 (3.71)	163 (5.08)		
Sugary beverages	0 times per week	448 (29.67)	481 (28.36)	929 (28.98)	39.303	0.000**
	1–2 times per week	628 (41.59)	803 (47.35)	1,431 (44.64)		
	3–5 times per week	193 (12.78)	247 (14.56)	440 (13.72)		
	6–7 times per week	110 (7.28)	97 (5.72)	207 (6.46)		
	≥8 times per week	131 (8.68)	68 (4.01)	199 (6.21)		
Snacks and desserts	0 times per week	401 (26.56)	308 (18.16)	709 (22.11)	55.680	0.000**
	1–2 times per week	548 (36.29)	781 (46.05)	1,329 (41.45)		
	3–5 times per week	324 (21.46)	361 (21.29)	685 (21.37)		
	6–7 times per week	131 (8.68)	172 (10.14)	303 (9.45)		
	≥8 times per week	106 (7.02)	74 (4.36)	180 (5.61)		
Fried foods	0 times per week	295 (19.54)	333 (19.63)	628 (19.59)	9.593	0.048*
	1–2 times per week	763 (50.53)	876 (51.65)	1,639 (51.12)		
	3–5 times per week	277 (18.34)	306 (18.04)	583 (18.18)		
	6–7 times per week	80 (5.30)	111 (6.54)	191 (5.96)		
	≥8 times per week	95 (6.29)	70 (4.13)	165 (5.15)		
Total		1,510	1,696	3,206		

* *p* < 0.05, ** *p* < 0.01.

### 3.3 Unprocessed or minimally processed foods

**Figure 2:**
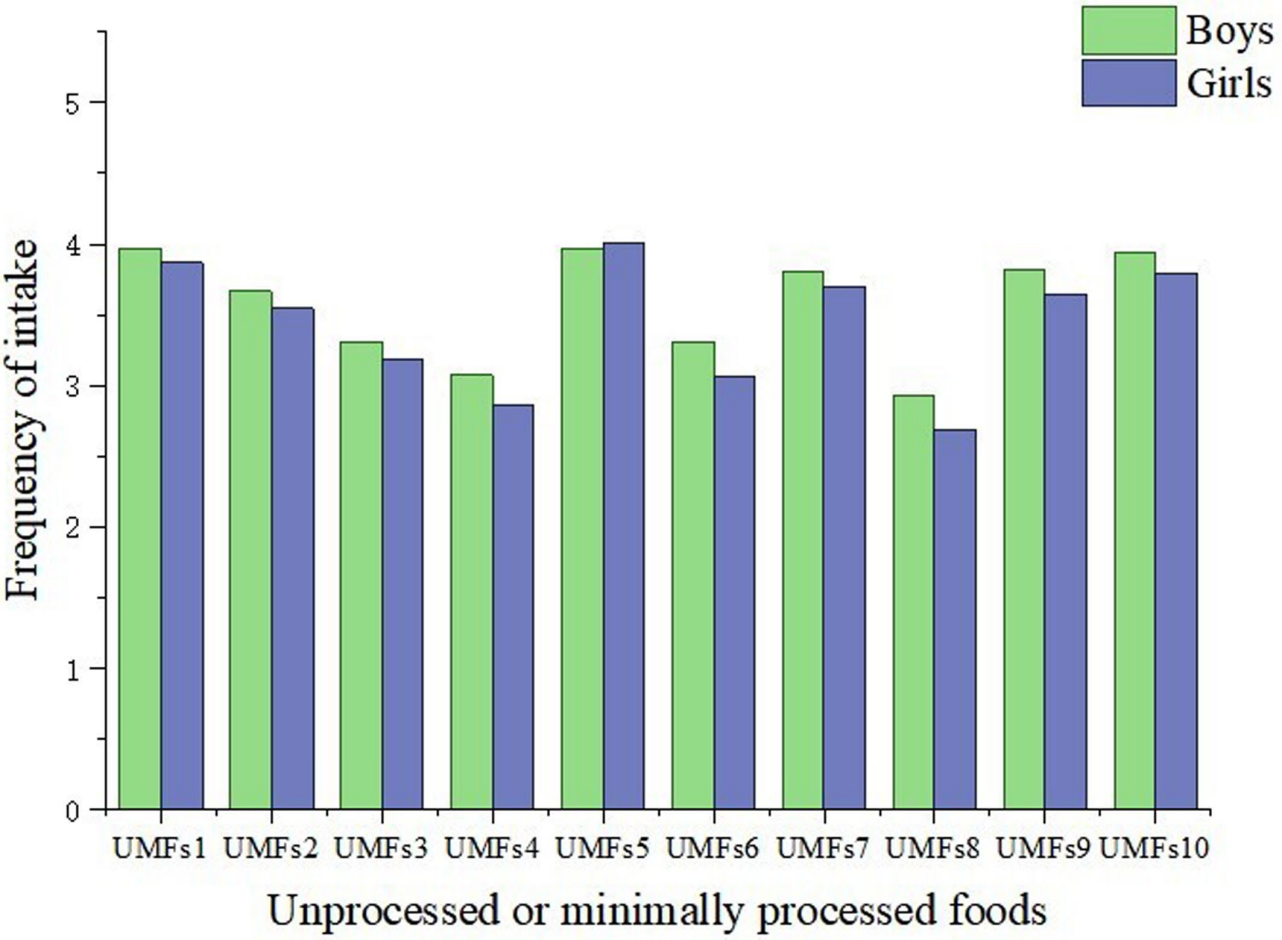
Gender differences in unprocessed or minimally processed foods intake in two waves of data (*N* = 3,206). UMFs1, green leafy vegetables; UMFs2, red and orange vegetables (such as carrots and tomatoes); UMFs3, potatoes (excluding fried potatoes, fried potato chips, or potato chips); UMFs4, tubers (such as sweet potatoes, yams, and taro); UMFs5, fruits; UMFs6, soy products (such as tofu and other soy-based products); UMFs7, fresh poultry, pork, beef, or other meats; UMFs8, fish, shrimp, or other seafood; UMFs9, eggs; UMFs10, dairy products.

Table 3 displays the intake of different types of UMFs among adolescents and their gender differences. For the entire student population, green leafy vegetables were the most popular, with about 75% of students consuming them at least 6–7 times per week. Fruits followed, with about 72% of students consuming them at least 6–7 times per week. Dairy products were the third most popular, with about 68% of students consuming them at least 6–7 times per week. Significant gender differences were found in all types of UMFs. Except for fruits, boys consumed different food types more than 8 times per week more frequently than girls. However, girls consumed fruits more frequently than boys. Additionally, 18% of girls had no intake of fish, shrimp, or seafood per week, a rate much higher than the 10% of boys. In addition, as shown in Figure 2, we visualized gender differences in UMFs intake.

**Table 3:** Intake of unprocessed or minimally processed food and gender differences of Chinese adolescents at follow-up (*N* = 3,206).

Item	Characteristics	Sex (%)		Total	χ2	*p*
		Boys	Girls			
Green leafy vegetables	0 times per week	11 (0.73)	14 (0.83)	25 (0.78)	16.906	0.002**
	1–2 times per week	112 (7.42)	123 (7.25)	235 (7.33)		
	3–5 times per week	228 (15.10)	321 (18.93)	549 (17.12)		
	6–7 times per week	472 (31.26)	577 (34.02)	1,049 (32.72)		
	≥8 times per week	687 (45.50)	661 (38.97)	1,348 (42.05)		
Red and orange vegetables (such as carrots and tomatoes)	0 times per week	26 (1.72)	30 (1.77)	56 (1.75)	35.752	0.000**
	1–2 times per week	241 (15.96)	234 (13.80)	475 (14.82)		
	3–5 times per week	411 (27.22)	625 (36.85)	1,036 (32.31)		
	6–7 times per week	399 (26.42)	411 (24.23)	810 (25.27)		
	≥8 times per week	433 (28.68)	396 (23.35)	829 (25.86)		
Potatoes (excluding fried potatoes, fried potato chips, or potato chips), and	0 times per week	72 (4.77)	46 (2.71)	118 (3.68)	32.566	0.000**
	1–2 times per week	375 (24.83)	462 (27.24)	837 (26.11)		
	3–5 times per week	468 (30.99)	636 (37.50)	1,104 (34.44)		
	6–7 times per week	285 (18.87)	283 (16.69)	568 (17.72)		
	≥8 times per week	310 (20.53)	269 (15.86)	579 (18.06)		
Tubers (such as sweet potatoes, yams, and taro)	0 times per week	148 (9.80)	193 (11.38)	341 (10.64)	50.860	0.000**
	1–2 times per week	468 (30.99)	653 (38.50)	1,121 (34.97)		
	3–5 times per week	350 (23.18)	404 (23.82)	754 (23.52)		
	6–7 times per week	241 (15.96)	246 (14.50)	487 (15.19)		
	≥8 times per week	303 (20.07)	200 (11.79)	503 (15.69)		
Fruits	0 times per week	20 (1.32)	25 (1.47)	45 (1.40)	16.227	0.003**
	1–2 times per week	145 (9.60)	116 (6.84)	261 (8.14)		
	3–5 times per week	287 (19.01)	290 (17.10)	577 (18.00)		
	6–7 times per week	502 (33.25)	543 (32.02)	1,045 (32.60)		
	≥8 times per week	556 (36.82)	722 (42.57)	1,278 (39.86)		
Soy products (such as tofu and other soy-based products)	0 times per week	83 (5.50)	80 (4.72)	163 (5.08)	105.247	0.000**
	1–2 times per week	295 (19.54)	523 (30.84)	818 (25.51)		
	3–5 times per week	506 (33.51)	650 (38.33)	1,156 (36.06)		
	6–7 times per week	316 (20.93)	247 (14.56)	563 (17.56)		
	≥8 times per week	310 (20.53)	196 (11.56)	506 (15.78)		
Fresh poultry, pork, beef, or other meats	0 times per week	25 (1.66)	29 (1.71)	54 (1.68)	30.467	0.000**
	1–2 times per week	152 (10.07)	177 (10.44)	329 (10.26)		
	3–5 times per week	427 (28.28)	514 (30.31)	941 (29.35)		
	6–7 times per week	390 (25.83)	540 (31.84)	930 (29.01)		
	≥8 times per week	516 (34.17)	436 (25.71)	952 (29.69)		
Fish, shrimp, or other seafood	0 times per week	145 (9.60)	301 (17.75)	446 (13.91)	100.443	0.000**
	1–2 times per week	537 (35.56)	633 (37.32)	1,170 (36.49)		
	3–5 times per week	412 (27.28)	507 (29.89)	919 (28.67)		
	6–7 times per week	200 (13.25)	122 (7.19)	322 (10.04)		
	≥8 times per week	216 (14.30)	133 (7.84)	349 (10.89)		
Eggs	0 times per week	20 (1.32)	37 (2.18)	57 (1.78)	51.826	0.000**
	1–2 times per week	141 (9.34)	244 (14.39)	385 (12.01)		
	3–5 times per week	389 (25.76)	442 (26.06)	831 (25.92)		
	6–7 times per week	498 (32.98)	617 (36.38)	1,115 (34.78)		
	≥8 times per week	462 (30.60)	356 (20.99)	818 (25.51)		
Dairy products	0 times per week	29 (1.92)	30 (1.77)	59 (1.84)	38.539	0.000**
	1–2 times per week	119 (7.88)	180 (10.61)	299 (9.33)		
	3–5 times per week	260 (17.22)	413 (24.35)	673 (20.99)		
	6–7 times per week	525 (34.77)	540 (31.84)	1,065 (33.22)		
	≥8 times per week	577 (38.21)	533 (31.43)	1,110 (34.62)		
Total		1,510	1,696	3,206		

* *p* < 0.05, ** *p* < 0.01.

### 3.4 The relationship between ultra-processed foods and quality of life

This longitudinal study used FE to examine the impact of UPF on the QoL among adolescents (shown in Table 4). Model 1 showed the relationship between UPF and QoL, while Model 2 added control variables. We observed that the UPF has negative effect to the QoL (All: *β* = −2.705, t = −4.52; Male: *β* = −3.480, t = −3.78; Female: *β* = −1.651, t = −2.19). In addition, we confirmed that instant noodles, sugary beverages, salty snacks or desserts, and fried foods had a negative impact on the QoL of adolescents, while processed meats and fast food had a positive impact. Additionally, there were gender differences in the effects of fast food and salty snacks or desserts on QoL. Specifically: (1) Processed meats had a significant positive effect on the QoL of all adolescents (All: *β* = 1.703, t = 4.28; Male: *β* = 2.128, t = 3.72; Female: *β* = 1.392, t = 2.49). (2) Instant noodles had a significant negative impact on the QoL of all adolescents (All: *β* = −2.696, t = −5.49; Male: β = −2.488, t = −3.48; Female: β = −3.698, t = −5.58). (3) Fast food had a significant positive effect on the QoL of girls, but no significant effect on the QoL of boys (All: *β* = 1.376, t = 2.47; Female: *β* = 2.306, t = 3.07). (4) The higher the frequency of sugary beverage consumption, the more detrimental it was to the QoL of all adolescents (All: β = −2.838, t = −5.29; Male: β = −1.933, t = −2.41; Female: β = −3.670, t = −5.22). (5) The higher the frequency of salty snack or dessert consumption, the worse the QoL of girls, but had no significant impact on boys (Female: β = −1.698, t = −2.55). (6) Fried foods were detrimental to the QoL of all adolescents (All: β = −2.796, t = −4.92; Male: β = −3.584, t = −4.26; Female: β = −1.492, t = −2.01).

**Table 4:** Results of fixed effects model analysis of ultra-processed foods and quality of life among Chinese adolescents in two waves of data (*N* = 3,206).

	Model 1			Model 2		
	All *β*	Male *β*	Female *β*	All *β*	Male *β*	Female *β*
UPF total score	−2.732***	−3.314***	−1.997**	−2.705***	−3.480***	−1.651*
	(−4.60)	(−3.58)	(−2.67)	(−4.52)	(−3.78)	(−2.19)
Processed meats	1.702***	2.083***	1.205**	1.703***	2.128***	1.392**
	(4.26)	(3.66)	(2.14)	(4.28)	(3.72)	(2.49)
Instant noodles	−2.755***	−2.374***	−3.415***	−2.696***	−2.488***	−3.698***
	(−5.61)	(−3.30)	(−5.10)	(−5.49)	(−3.48)	(−5.58)
Western fast food	1.462***	−0.204	2.920***	1.376**	0.095	2.306***
	(2.66)	(−0.25)	(3.89)	(2.47)	(0.12)	(3.07)
Sugary beverages	−2.750***	−1.804**	−3.635***	−2.838***	−1.933**	−3.670***
	(−5.11)	(−2.22)	(−5.12)	(−5.29)	(−2.41)	(−5.22)
Snacks and desserts	−0.805	0.325	−1.820***	−0.797	0.438	−1.698**
	(−1.59)	(0.42)	(−2.72)	(−1.57)	(0.57)	(−2.55)
Fried foods	−2.813***	−3.539***	−1.786**	−2.796***	−3.584***	−1.492**
	(−4.96)	(−4.17)	(−2.41)	(−4.92)	(−4.26)	(−2.01)
Age				−0.460	−2.877***	1.859***
				(−0.91)	(−3.87)	(2.72)
SES				0.113**	−0.086	0.333***
				(2.11)	(−1.12)	(4.57)
Constant	157.209***	156.671***	158.197***	161.380***	198.845***	123.743***
	(126.66)	(88.03)	(91.78)	(21.82)	(18.86)	(12.17)
*N*	3,206	1,510	1,696	3,206	1,510	1,696
R2	0.080	0.077	0.095	0.082	0.088	0.111
adj. R2	0.0795	0.0753	0.0930	0.0809	0.0856	0.109

*** *p* < 0.01, ** *p* < 0.05, * *p* < 0.1. Robust *t*-statistics in parentheses; UPF, ultra-processed foods.

### 3.5 The relationship between unprocessed or minimally processed foods and quality of life

Afterward, using the two waves of data, we examined the impact of UMFs on the QoL among adolescents (shown in Table 5). Model 2 included control variables based on Model 1. After controlling for some variables, although the total UMFs score did not have a significant effect on QoL, we found that, except for eggs and dairy products, the other types of UMFs had an impact on the QoL of adolescents. Among these, green leafy vegetables, red and orange vegetables (such as carrots and tomatoes), fruits, fresh poultry, pork, beef, or other meats, and fish, shrimp, or other seafood had a positive impact on adolescent QoL, while potatoes (excluding fried potatoes, fried potato chips, or potato chips), tubers (such as sweet potatoes, yams, and taro), and soy products (such as tofu and other soy-based products) had a negative impact.

**Table 5:** Results of fixed effects model analysis of between unprocessed or minimally processed foods and quality of life among Chinese adolescents in two waves of data (*N* = 3,206).

	Model 1			Model 2		
	All *β*	Male *β*	Female *β*	All *β*	Male *β*	Female *β*
UMFs total score	0.550	0.270	0.903	0.578	0.215	0.967
	(1.24)	(0.43)	(1.42)	(1.29)	(0.34)	(1.51)
Green leafy vegetables	1.470***	1.640**	1.479**	1.518***	0.835	2.109***
	(3.16)	(2.20)	(2.47)	(3.01)	(1.05)	(3.25)
Red and orange vegetables (such as carrots and tomatoes)	0.916*	0.135	1.689***	0.917*	0.421	1.758***
	(1.95)	(0.20)	(2.67)	(1.95)	(0.61)	(2.79)
Potatoes (excluding fried potatoes, fried potato chips, or potato chips)	−2.359***	−1.627***	−2.979***	−2.358***	−1.494***	−3.012***
	(−5.96)	(−2.84)	(−5.42)	(−5.97)	(−2.62)	(−5.52)
Tubers (such as sweet potatoes, yams, and taro)	−0.605	−1.358**	0.001	−0.603	−1.387**	−0.008
	(−1.32)	(−2.11)	(0.00)	(−1.32)	(−2.17)	(−0.01)
Fruits	2.190***	3.597***	1.031*	2.196***	3.715***	1.123*
	(4.87)	(5.25)	(1.75)	(4.87)	(5.47)	(1.85)
Soy products (such as tofu and other soy-based products)	−1.198***	−0.255	−2.154***	−1.202***	−0.443	−2.069***
	(−2.59)	(−0.37)	(−3.49)	(−2.60)	(−0.64)	(−3.34)
Fresh poultry, pork, beef, or other meats	0.821*	1.210*	0.626	0.823*	1.159*	0.646
	(1.83)	(1.80)	(1.04)	(1.84)	(1.73)	(1.08)
Fish, shrimp, or other seafood	1.827***	0.428	2.900***	1.817***	0.694	2.438***
	(4.36)	(0.72)	(4.88)	(4.29)	(1.16)	(4.08)
Eggs	0.346	0.786	−0.126	0.312	1.092	−0.491
	(0.65)	(0.94)	(−0.18)	(0.57)	(1.29)	(−0.68)
Dairy products	0.603	0.306	0.956	0.617	0.232	1.004
	(1.36)	(0.49)	(1.50)	(1.38)	(0.37)	(1.57)
Age				0.160	−2.647***	2.462***
				(0.28)	(−3.24)	(3.16)
SES				−0.007	−0.113	0.159**
				(−0.13)	(−1.43)	(2.07)
Constant	122.058***	118.741***	124.917***	119.854***	159.131***	85.403***
	(55.39)	(36.29)	(41.46)	(14.16)	(13.42)	(7.07)
N	3,206	1,510	1,696	3,206	1,510	1,696
R2	0.065	0.075	0.072	0.065	0.085	0.082
adj. R2	0.0638	0.0720	0.0694	0.0635	0.0809	0.0781

*** *p* < 0.01, ** *p* < 0.05, * *p* < 0.1. Robust *t*-statistics in parentheses. UMFs, unprocessed or minimally processed foods.

We also found gender differences in these effects: (1) Green leafy vegetables had a positive effect on the QoL of girls (All: *β* = 1.518, t = 3.01; Female: β = 2.109, t = 3.25). (2) Red and orange vegetables (such as carrots and tomatoes) could promote the QoL of girls (All: *β* = 0.917, t = 1.95; Female: β = 1.758, t = 2.79). (3) Potatoes (excluding fried potatoes, fried potato chips, or potato chips) had a negative effect on the QoL of adolescents (All: *β* = −2.358, t = −5.97; Male: *β* = −1.494, t = −2.62; Female: *β* = −3.012, t = −5.52). (4) The frequency of fruit intake helped promote the QoL of adolescents (All: *β* = 2.196, t = 4.87; Male: *β* = 3.715, t = 5.47; Female: *β* = 1.123, t = 1.85). (5) Soy products were detrimental to the QoL of girls (All: *β* = −1.202, t = −2.60; Female: *β* = −2.069, t = −3.34). (6) Fresh poultry, pork, beef, or other meats had a positive effect on the QoL of boys (All: *β* = 0.823, t = 1.84; Male: *β* = 1.159, t = 1.73). (7) Fish, shrimp, or other seafood had a positive effect on the QoL of girls (All: *β* = 1.817, t = 4.29; Female: *β* = 2.438, t = 4.08). (8) Tubers (such as yams, taro, sweet potatoes, etc.) had a negative effect on the QoL of boys (Male: *β* = −1.387, t = −2.17).

## 4 Discussion

Through a two-year longitudinal survey of adolescents, this study used FE to analyze the impact of intake frequencies of UMFs and UPF on the QoL of 3,206 adolescents, and to explore gender differences. The findings of the study indicate: (1) Boys consumed UPF at significantly higher frequencies than girls. (2) Except for fruits, boys consumed UMFs more frequently than girls. (3) Girls had lower QoL compared to boys. (4) Not all UPF had a negative impact on the QoL of adolescents, and there were gender differences in these effects. (5) Different UMFs had varying impacts on the QoL of adolescents, and there were gender differences in these effects.

### 4.1 The gender differences in ultra-processed foods and unprocessed or minimally processed foods intake

We found that there are gender differences in dietary intake frequency (31). Specifically, from the point of UPF, the findings that boys tend to consume more processed meats, instant noodles, fast food, sugary beverages, desserts, salty snacks, and fried foods than girls align with some previous studies. A study on Taiwanese adolescents, confirmed that boys appear to consume fast food, high-fat snacks, processed meats, and sugary beverages more frequently (32). From the point of UMFs, girls consume fruits more frequently than boys, but all other types of UMFs were lower than boys (33). Previous studies have also reported that school-age boys prefer meat and girls prefer fruits and vegetables (34).

We believe that there are several potential reasons for these differences. Firstly, from the perspective of psychology mechanism, girls are more likely to be influenced by external social factors such as higher satisfaction with appearance and appearance anxiety (35), leading to differences in dietary choices compared to boys (36). Secondly, from the perspective of physiological mechanism, smell, BMI, sex hormones, menstrual cycle are all important factors that affect women’s food preferences (37). Thirdly, from a sociological point of view, social gatherings, parents’ eating habits, food advertising and marketing are also direct causes of adolescents (38).

### 4.2 The impact of ultra-processed foods on quality of life

We found that processed meats have a significant positive effect on the QoL of all adolescents. This result differs from the majority of previous studies that have shown an association between processed meats and increased disease risk (39), as well as a link between processed meats and an increased incidence of dental caries in adolescents (40). The potential reasons for this discrepancy may include the emotional preferences of Chinese adolescents for processed meats. Since QoL measures include a variety of psychological aspects, the higher psychological scores of adolescents could influence the assessment of QoL.

The findings that instant noodles, sugary beverages, and fried foods have a negative impact on the QoL of all adolescents are consistent with our expectations. A study on Japanese adolescents, for example, has shown that frequent consumption of instant noodles is associated with increased dental caries (40), shorter sleep duration, and poor sleep quality (41). The safety of frequent instant noodle consumption among adolescents is also a concern, as reports indicate a high incidence of burns due to instant noodle incidents each year (42, 43).

Consistent with our findings, frequent sugary beverage intake among adolescents has been linked to shorter sleep duration (41), poor sleep quality (41, 44), lower muscle strength (45), higher BMI (46), higher serum uric acid levels and blood pressure (47), poorer diet quality (48), greater stress, stronger suicidal ideation, and higher rates of depression (49). These factors can contribute to adverse health outcomes in adolescents.

The negative effects of fried foods on adolescents should not be underestimated. Fried foods like French fries and fried chicken are popular among students (50), but pose significant health risks due to their high saturated fat and sodium content (51), including the risk of overweight, obesity, impaired glucose tolerance (29), elevated blood pressure (52), and cardiovascular disease (53). Moreover, the processing method often leads to the formation of acrylamide (54), a substance classified as a carcinogen (55). Additionally, fried foods can lead to the formation of polycyclic aromatic hydrocarbons (PAHs), which also pose a potential cancer risk (56).

The study results also show that fast food has a significant positive impact on the QoL of girls but not on the boys. In China, fast food mainly comes from sources like KFC, McDonald’s, and Pizza Hut (57). One possible explanation is that for adolescents, eating fast food appears to be a way to socialize with family and friends, and they think fast food has a good taste (58). Scholars have also explained from a sociological perspective that unhealthy food is an important social element for adolescents, rather than a component of daily consumption, and sharing food with peers can promote a sense of belonging to a group (59). It all seems to have to do with improved QoL for adolescents. However, some previous research findings suggest that from a physical perspective, fast food due to its high energy density and glycemic load, contributes to increased rates of overweight (60), short sleep duration, poor sleep quality (41), and dental caries (40). Psychologically, fast food intake is associated with greater stress, stronger suicidal ideation, and higher rates of depression (49). However, some studies have questioned these results, with some scholars finding no significant association between fast food consumption and BMI in children (61), and no correlation with fat status (62), nor any association between fast food intake and obesity, central obesity, and hypertension (63). In general, the existing results are mixed, but we believe that the consumption of fast food in a controlled range will not reduce the QoL of adolescents, and moderate consumption of fast food is appropriate.

Finally, it is worth noting that the higher the frequency of salt-added snack or dessert intake, the worse the QoL of girls, but this had no significant effect on boys. This gender difference can be observed in the descriptive analysis, where significantly more boys consume no snacks or desserts per week (about 27%) than girls (about 18%). The higher fluctuating levels of sex hormones in women during adolescence are one of the reasons why they choose more desserts and snacks (64). The menstrual cycle also had a greater impact on their food preferences (37). However, it cannot be denied that salt-added snacks and desserts have a negative impact on the QoL of adolescents, which can not only affect sleep duration and quality (41), but also cause adverse health outcomes such as hypertension (65). In addition, adolescents under greater stress are more likely to consume more snacks or desserts (66). These factors could potentially lead to a lower QoL for adolescents.

In summary, we recommend strengthening the attention paid to the intake of UPF among adolescents. Healthy foods are becoming more expensive (67), and UPF has an increasingly consumption rate among adolescents due to its easy availability, cheap, and ready-to-eat (68). Adolescents are also influenced by the marketing of UPF and have a direct effect on food choice and consumption (69). We must intervene in this phenomenon and all sectors of society should provide an environment with better healthy food choices for adolescents (70), such as subsidies for vegetables and fruits, taxes on fast food, restrictions on the commercialization of UPF in schools, and improving supermarket access for teenagers (67).

### 4.3 The impact of unprocessed or minimally processed foods on quality of life

Fruit intake has a positive impact on the QoL of all adolescents. Green leafy vegetables, red and orange vegetables (such as carrots, tomatoes, etc.), and fish/shrimp or other seafood have a positive impact on the QoL of girls. Fresh poultry, pork, beef, etc., have a positive effect on the QoL of boys. Soy products, however, are detrimental to the QoL of girls. Tubers (such as yams, taro, sweet potatoes, etc.) have a negative effect on the QoL of boys. Potatoes have a negative effect on the QoL of all adolescents. We also found that fish/shrimp or other seafood has a positive effect on girls’ QoL.

The potential reasons for gender differences could be due to the strong influence of personal motivation on vegetable intake (71). For example, we found that green leafy vegetables and red and orange vegetables can improve girls’ QoL, but not significantly in boys. Some scholars have reported that girls have a higher preference for vegetables than boys (72). Therefore, compared to boys, girls’ vegetable intake has a more significant impact on them. Consequently, compared to boys, girls’ intake of vegetables has a more significant impact. Vegetables have been proven to have profound health-promoting effects, including the reduction of blood pressure (73), cardiovascular health (74), abdominal obesity, triglycerides, and low-density lipoprotein cholesterol (75), and the mitigation of the risk of metabolic syndrome (76) when consumed daily.

Recent studies have reported that seafood intake can reduce the risk of obesity in adolescents (35). Fish/shrimp or other seafood is an important dietary source of omega-3 long-chain polyunsaturated fatty acids, and Omega-3 long-chain polyunsaturated fatty acids are essential nutrients for the healthy development (77, 78). Additionally, seafood consumption helps reduce the lifetime prevalence of bipolar affective disorder (79). However, seafood can cause allergic reactions in some individuals, so we also suggest being cautious about seafood intake based on personal circumstances and avoiding adverse physical reactions (80). It is worth noting that our study found no effect of these foods on QoL in boys. One possible explanation is that, a study of Chinese adolescents found that children of fathers with less education had lower seafood consumption preferences (81). Our descriptive analysis also shows that boys’ fathers are less educated than girls’ fathers.

Fruits are beneficial for the QoL of all adolescents. Our results once again validate previous studies showing that fruit intake frequency helps to avoid negative mental health issues (82), promotes better well-being (83), and lowers the incidence of depression (84). A high intake of fruits is strongly associated with overall health, oral health, and satisfaction with sleep (85).

Fresh poultry, pork, beef, and other meats have a positive impact on the QoL of boys. Studies have reported that fresh beef and lamb are rich in protein, monounsaturated fats, vitamin D, B12, niacin, iron, and zinc, with less fat, saturated fat, and salt (86). Consuming poultry helps achieve adequate iron intake to prevent anemia (87). Pork is a good source of protein and can improve body composition (88).

It is worth noting that we observed that legume foods have an adverse effect on the QoL of adolescents. This differs from some studies that report that a diet containing legumes helps improve dietary quality scores, and may help reduce BMI (89), weight, body fat percentage, improve low-density lipoprotein cholesterol, and waist circumference (90, 91). In contrast, some scholars have found that the intake of snacks leads to adolescents with overweight or obesity consuming a large amount of grain, tubers, and legumes, which results in higher carbohydrate intake, and has confirmed that grain, tuber, and legume intake is associated with overweight/obesity in children and adolescents (92). This may be a potential reason for the reduced QoL of adolescents.

Similarly, we found that potatoes and tubers such as yams, taro, and sweet potatoes are detrimental to the QoL of adolescents. The impact of potatoes and other starchy vegetables on health is controversial. Studies have shown that potato intake may increase waist circumference (93), and starchy vegetables do not significantly reduce the risk of metabolic syndrome in adolescents compared to green leafy vegetables (76). This finding provides a basis for further exploration, and it is important to understand the intrinsic factors that lead to these differences in the future.

In summary, our study found that many UMFs have a positive effect on the QoL of adolescents, while most UPF have an adverse effect. Adolescents seem to prefer the taste of UPF (94) and they occupy a higher proportion in the overall diet (24). Therefore, cultivating and intervening in students’ eating habits are effective ways to improve QoL. Since the diet patterns of adolescence can extend into adulthood (95), changing unhealthy eating behaviors early in life is important (73). The WHO advocates for a diet rich in fruits and vegetables as a key component of a healthy diet (96). We have the same call to reduce the intake of UPF and increase the intake of UMFs in adolescents to help improve their QoL (25).

### 4.4 Limitation

While this study provides new insights and perspectives on the diets and QoL of Chinese adolescents, and conducts differential analysis considering intrinsic factors, it is important to mention the limitations of this research. Firstly, due to China’s unique eating habits, the higher sodium content should be considered in future research. Secondly, in addition to SES, gender, and age, there are many potential confounding factors that may affect food choices and intake should be considered (81, 97), such as parental eating habits, the influence of commercial advertisements (98), illness severity, participation in physical activities, and screen time use (38). Specifically, parental eating habits have a direct impact on the diets of adolescents; there are dietary differences between adolescents with and without illnesses; adolescents who are highly active in sports may have inconsistent dietary habits compared to others; and adolescents with a BMI in the overweight or obese range may have different dietary or QoL patterns due to stigmatization. Therefore, these potential factors should be considered in future research to gain a more comprehensive understanding of the relationship between diet and QoL in adolescents.

## 5 Conclusion

Our findings reveal that various types of UMFs, as well as UPF, yield discrepant outcomes on the QoL among adolescents, and such effects exhibit gender disparities. (1) Instant noodles, sugary beverages, and fried foods have been identified as having a detrimental impact on adolescents. (2) Snacks or desserts adversely affect the QoL only in girls. (3) Processed meats, however, contribute positively to the QoL for all adolescents. (4) Fast food is beneficial for girls’ QoL. (5) Fruits have a positive impact on the QoL for all adolescents. (6) Green leafy vegetables, as well as red and orange vegetables such as carrots and tomatoes, have a positive influence on girls’ QoL. (7) Fresh poultry, pork, beef, and the like positively affect boys’ QoL. (8) Conversely, soy-based products are found to be detrimental to girls’ QoL. (9) Tubers, including yams, taro, and sweet potatoes, have a negative impact on boys’ QoL. (10) Potatoes, in general, have a detrimental effect on the QoL for all adolescents. These discoveries are of profound significance for the health of adolescents. This study draws attention to dietary interventions that can be employed to improve the QoL of adolescents. Our results offer new insights and approaches for the intervention and enhancement of the QoL among Chinese adolescents.

## Data Availability

The datasets presented in this study can be found in online repositories. The names of the repository/repositories and accession number(s) can be found at: Database of Youth Health [https://www.ncmi.cn//phda/dataDetails.do?id=CSTR:17970.11.A0031.202107.209.V1.0].

## References

[1] World Health Organization (2024) WHOQOL-measuring quality of life. Available at: https://www.who.int/tools/whoqol (accessed august 13, 2024).

[2] Guan KW Adlung C Keijsers L Smit CR Vreeker A Thalassinou E. Just-in-time adaptive interventions for adolescent and young adult health and well-being: protocol for a systematic review. BMJ Open. (2024) 14:e083870. doi: 10.1136/bmjopen-2024-083870, PMID: 38955365 PMC11218018

[3] Magai DN Koot HM. Quality of life in children and adolescents in Central Kenya: associations with emotional and behavioral problems. Qual Life Res. (2019) 28:1271–9. doi: 10.1007/s11136-019-02099-8 30656535 PMC6470276

[4] Wallander JL Koot HM. Quality of life in children: a critical examination of concepts, approaches, issues, and future directions. Clin Psychol Rev. (2016) 45:131–43. doi: 10.1016/j.cpr.2015.11.007, PMID: 26911191

[5] Global nutrition targets (2025) Stunting policy brief. Available at: https://www.who.int/publications/i/item/WHO-NMH-NHD-14.3 (accessed august 11, 2024).

[6] Sawyer SM. Global growth trends in school-aged children and adolescents. Lancet. (2020) 396:1465–7. doi: 10.1016/S0140-6736(20)32232-7, PMID: 33160551

[7] Global nutrition targets (2025) Childhood overweight policy brief. Available at: https://www.who.int/publications/i/item/WHO-NMH-NHD-14.6 (accessed august 11, 2024).

[8] Dehghan M Akhtar-Danesh N Merchant AT. Childhood obesity, prevalence and prevention. Nutr J. (2005) 4:24. doi: 10.1186/1475-2891-4-24, PMID: 16138930 PMC1208949

[9] Rodriguez-Martinez A Zhou B Sophiea MK Bentham J Paciorek CJ Iurilli ML. Height and body-mass index trajectories of school-aged children and adolescents from 1985 to 2019 in 200 countries and territories: a pooled analysis of 2181 population-based studies with 65 million participants. Lancet. (2020) 396:1511–24. doi: 10.1016/S0140-6736(20)31859-6, PMID: 33160572 PMC7658740

[10] Govindaraju T Sahle BW McCaffrey TA McNeil JJ Owen AJ. Dietary patterns and quality of life in older adults: a systematic review. Nutrients. (2018) 10:971. doi: 10.3390/nu10080971, PMID: 30050006 PMC6115962

[11] Hosseininasab D Shiraseb F Bahrampour N da Silva A Hajinasab MM Bressan J. Ultra-processed food consumption and quality of life: a cross-sectional study in Iranian women. Front *Public Health*. (2024) 12:1351510. doi: 10.3389/fpubh.2024.1351510, PMID: 38665244 PMC11043594

[12] Diao H Wang H Yang L Li T. The impacts of multiple obesity-related interventions on quality of life in children and adolescents: a randomized controlled trial. Health Qual Life Outcomes. (2020) 18:213. doi: 10.1186/s12955-020-01459-0, PMID: 32631401 PMC7336614

[13] Ng L-H Hart M Dingle SE Milte CM Livingstone KM Shaw JE. Prospective associations between diet quality and health-related quality of life in the Australian diabetes, obesity and lifestyle (AusDiab) study. Br J Nutr. (2023) 130:83–92. doi: 10.1017/S000711452200304X, PMID: 36128619

[14] Ramin S Mysz MA Meyer K Capistrant B Lazovich D Prizment A. A prospective analysis of dietary fiber intake and mental health quality of life in the Iowa Women’s health study. Maturitas. (2020) 131:1–7. doi: 10.1016/j.maturitas.2019.10.007, PMID: 31787141 PMC6916712

[15] Şimşek H Uçar A. Nutritional status and quality of life are associated with risk of sarcopenia in nursing home residents: a cross-sectional study. Nutr Res. (2022) 101:14–22. doi: 10.1016/j.nutres.2022.02.002 35358793

[16] Chaumont S Quinquis L Monnerie B Six C Hébel P Chassany O. A poor diet quality is associated with more gas-related symptoms and a decreased quality of life in French adults. Br J Nutr. (2023) 129:715–24. doi: 10.1017/S0007114522001593, PMID: 35603426 PMC9899566

[17] Zhang J Wang Z Du W Huang F Jiang H Bai J. Twenty-five-year trends in dietary patterns among Chinese adults from 1991 to 2015. Nutrients. (2021) 13:1327. doi: 10.3390/nu13041327, PMID: 33923855 PMC8072541

[18] Pan F Zhang T Mao W Zhao F Luan D Li J. Ultra-processed food consumption and risk of overweight or obesity in Chinese adults: Chinese food consumption survey 2017–2020. Nutrients. (2023) 15:4005. doi: 10.3390/nu15184005, PMID: 37764788 PMC10537323

[19] Pan X-F Wang L Pan A. Epidemiology and determinants of obesity in China. Lancet Diabetes Endocrinol. (2021) 9:373–92. doi: 10.1016/S2213-8587(21)00045-0, PMID: 34022156

[20] Wang H Sekine M Chen X Yamagami T Kagamimori S. Lifestyle at 3 years of age and quality of life (QOL) in first-year junior high school students in Japan: results of the Toyama birth cohort study. Qual Life Res. (2008) 17:257–65. doi: 10.1007/s11136-007-9301-6, PMID: 18157615

[21] Hendriksen RB van der Gaag EJ. Effect of a dietary intervention including minimal and unprocessed foods, high in natural saturated fats, on the lipid profile of children, pooled evidence from randomized controlled trials and a cohort study. PLoS One. (2022) 17:e0261446. doi: 10.1371/journal.pone.0261446 34986194 PMC8730453

[22] Zeng H Wang B Zhang R Zhao L Yang Y Dong X. Association of parent-child discrepancies in educational aspirations with physical fitness, quality of life and school adaptation among adolescents: a multiple mediation model. BMC Public Health. (2024) 24:2135. doi: 10.1186/s12889-024-19674-5, PMID: 39107725 PMC11304611

[23] Shengfa Z Wei L Xiaosheng D Wenxin C Xiangren Y Wei Z. A dataset on the status quo of health and health-related behaviors of Chinese youth: a longitudinal large-scale survey in the secondary school students of Shandong Province. Chin Med Sci J. (2022) 37:60–6. doi: 10.24920/004051, PMID: 35172918

[24] Melo AS Neves FS Batista AP Machado-Coelho GLL Sartorelli DS De FER. Percentage of energy contribution according to the degree of industrial food processing and associated factors in adolescents (EVA-JF study, Brazil). Public Health Nutr. (2021) 24:4220–9. doi: 10.1017/S1368980021000100, PMID: 33436138 PMC10195385

[25] Moubarac J-C Batal M Louzada ML Martinez Steele E Monteiro CA. Consumption of ultra-processed foods predicts diet quality in Canada. Appetite. (2017) 108:512–20. doi: 10.1016/j.appet.2016.11.006 27825941

[26] Monteiro CA Cannon G Moubarac J-C Levy RB Louzada MLC Jaime PC. The UN decade of nutrition, the NOVA food classification and the trouble with ultra-processing. Public Health Nutr. (2018) 21:5–17. doi: 10.1017/S1368980017000234, PMID: 28322183 PMC10261019

[27] Zou L Zhu K Jiang Q Xiao P Wu X Zhu B. Quality of life in Chinese children with developmental dyslexia: a cross-sectional study. BMJ Open. (2022) 12:e052278. doi: 10.1136/bmjopen-2021-052278, PMID: 35039286 PMC8765030

[28] Hong X Liu Q Zhao S. Approaches to learning of preschool children in China: a comparison between only children and non-only children. Behav Sci. (2023) 13:418. doi: 10.3390/bs13050418, PMID: 37232654 PMC10215645

[29] Wang VH Min J Xue H Du S Xu F Wang H. What factors may contribute to sex differences in childhood obesity prevalence in China? Public Health Nutr. (2018) 21:2056–64. doi: 10.1017/S1368980018000290, PMID: 29478427 PMC6062478

[30] Skårdal M Western IM Ask AMS Øverby NC. Socioeconomic differences in selected dietary habits among Norwegian 13–14 year-olds: a cross-sectional study. Food Nutr Res. (2014) 58:23590. doi: 10.3402/fnr.v58.23590, PMID: 25140123 PMC4111874

[31] Otsuka Y Kaneita Y Itani O Jike M Osaki Y Higuchi S. Gender differences in dietary behaviors among Japanese adolescents. Prev Med Rep. (2020) 20:101203. doi: 10.1016/j.pmedr.2020.101203, PMID: 32995146 PMC7509230

[32] Bui C Lin L-Y Wu C-Y Chiu Y-W Chiou H-Y. Association between emotional eating and frequency of unhealthy food consumption among Taiwanese adolescents. Nutrients. (2021) 13:2739. doi: 10.3390/nu13082739, PMID: 34444899 PMC8401002

[33] Wuenstel JW Wądołowska L Słowińska MA Niedźwiedzka E Kowalkowska J Kurp L. Intake of dietary fibre and its sources related to adolescents’ age and gender, but not to their weight. Cent Eur J Public Health. (2016) 24:211–6. doi: 10.21101/cejph.a4331 27743515

[34] Caine-Bish NL Scheule B. Gender differences in food preferences of school-aged children and adolescents. J Sch Health. (2009) 79:532–40. doi: 10.1111/j.1746-1561.2009.00445.x, PMID: 19840230

[35] Zhang T Ye H Pang X Liu X Hu Y Wang Y. Seafood intake in childhood/adolescence and the risk of obesity: results from a Nationwide cohort study. Nutr J. (2024) 23:77. doi: 10.1186/s12937-024-00986-6, PMID: 39010085 PMC11251353

[36] Oellingrath IM Hestetun I Svendsen MV. Gender-specific association of weight perception and appearance satisfaction with slimming attempts and eating patterns in a sample of young Norwegian adolescents. Public Health Nutr. (2016) 19:265–74. doi: 10.1017/S1368980015001007, PMID: 25866130 PMC10271024

[37] Hartman-Petrycka M Witkoś J Lebiedowska A Błońska-Fajfrowska B. Individual characteristics, including olfactory efficiency, age, body mass index, smoking and the sex hormones status, and food preferences of women in Poland. PeerJ. (2022) 10:e13538. doi: 10.7717/peerj.13538, PMID: 35726259 PMC9206430

[38] Rodríguez-Barniol M Pujol-Busquets G Bach-Faig A. Screen time use and ultra-processed food consumption in adolescents: a focus group qualitative study. J Acad Nutr Diet. (2024) 124:1336–46. doi: 10.1016/j.jand.2024.04.015, PMID: 38697354

[39] O’Connor LE Wambogo EA Herrick KA Parsons R Reedy J. A standardized assessment of processed red meat and processed poultry intake in the US population aged ≥2 years using NHANES. J Nutr. (2022) 152:190–9. doi: 10.1093/jn/nxab316, PMID: 34718661 PMC8754567

[40] da Silva NRJ de Camargo MBJ dos Vaz JS Correa MB Matijasevich A Da Silva dos Santos I. Ultra-processed food consumption and dental caries in adolescents from the 2004 Pelotas birth cohort study. Community Dent Oral Epidemiol. (2023) 51:1180–6. doi: 10.1111/cdoe.12851 37032457

[41] Min C Kim H-J Park I-S Park B Kim J-H Sim S. The association between sleep duration, sleep quality, and food consumption in adolescents: a cross-sectional study using the Korea youth risk behavior web-based survey. BMJ Open. (2018) 8:e022848. doi: 10.1136/bmjopen-2018-022848, PMID: 30042149 PMC6059317

[42] Koltz PF Wasicek P Mays C Bell DE. An unsuspected cause of meal-time morbidity: instant noodle scald burns. J Burn Care Res. (2013) 34:e244–9. doi: 10.1097/BCR.0b013e318270094f, PMID: 23202878

[43] Wu C Tan AL Maze DAE Holland AJA. Instant hot noodles: do they need to burn? Burns. (2013) 39:363–8. doi: 10.1016/j.burns.2012.06.005, PMID: 22975407

[44] Vézina-Im L-A Beaulieu D Turcotte S Turcotte A-F Delisle-Martel J Labbé V. Association between beverage consumption and sleep quality in adolescents. Nutrients. (2024) 16:285. doi: 10.3390/nu16020285, PMID: 38257178 PMC10819752

[45] Zhang Y Xu P Song Y Ma N Lu J. Association between sugar-sweetened beverage consumption frequency and muscle strength: results from a sample of Chinese adolescents. BMC Public Health. (2023) 23:1010. doi: 10.1186/s12889-023-15987-z, PMID: 37254093 PMC10227785

[46] Tobiassen PA-S Køster-Rasmussen R. Substitution of sugar-sweetened beverages with non-caloric alternatives and weight change: a systematic review of randomized trials and meta-analysis. Obes Rev. (2024) 25:e13652. doi: 10.1111/obr.13652 37880814

[47] Nguyen S Choi HK Lustig RH Hsu C. Sugar-sweetened beverages, serum uric acid, and blood pressure in adolescents. J Pediatr. (2009) 154:807–13. doi: 10.1016/j.jpeds.2009.01.015, PMID: 19375714 PMC2727470

[48] Fontes AS Pallottini AC Vieira DAS Batista LD Fontanelli MM Fisberg RM. Increased sugar-sweetened beverage consumption is associated with poorer dietary quality: a cross-sectional population-based study. Rev Nutr. (2019) 32:e180121. doi: 10.1590/1678-9865201932e180121

[49] Ra JS. Consumption of sugar-sweetened beverages and fast foods deteriorates adolescents’ mental health. Front Nutr. (2022) 9:1058190. doi: 10.3389/fnut.2022.1058190, PMID: 36618694 PMC9817134

[50] Islam S Rayna SE Khan FA Rahman KMT Piyal MSM Saha BK. Health compromising components in French fries and fried chicken available in the markets of Dhaka city, Bangladesh. CyTA J Food. (2023) 21:580–5. doi: 10.1080/19476337.2023.2257777

[51] Qin P Liu D Wu X Zeng Y Sun X Zhang Y. Fried-food consumption and risk of overweight/obesity, type 2 diabetes mellitus, and hypertension in adults: a meta-analysis of observational studies. Crit Rev Food Sci Nutr. (2021) 62:6809–20. doi: 10.1080/10408398.2021.1906626, PMID: 33825582

[52] Ooi DSQ Toh JY Ng LYB Peng Z Yang S Rashid NSBSA. Dietary intakes and eating behavior between metabolically healthy and unhealthy obesity phenotypes in Asian children and adolescents. Nutrients. (2022) 14:4796. doi: 10.3390/nu14224796, PMID: 36432482 PMC9697734

[53] Chen L Zhu H Gutin B Race DY. Gender, family structure, socioeconomic status, dietary patterns, and cardiovascular health in adolescents. Curr Dev Nutr. (2019) 3:nzz117. doi: 10.1093/cdn/nzz117, PMID: 31750413 PMC6856469

[54] Matthys C Bilau M Govaert Y Moons E De Henauw S Willems JL. Risk assessment of dietary acrylamide intake in Flemish adolescents. Food Chem Toxicol. (2005) 43:271–8. doi: 10.1016/j.fct.2004.10.003, PMID: 15621340

[55] INCHEM Acrylamide (IARC Summary & Evaluation, volume 60), (1994). Available at: https://inchem.org/documents/iarc/vol60/m60-11.html (accessed August 14, 2024).

[56] Li G Wu S Wang L Akoh CC. Concentration, dietary exposure and health risk estimation of polycyclic aromatic hydrocarbons (PAHs) in youtiao, a Chinese traditional fried food. Food Control. (2016) 59:328–36. doi: 10.1016/j.foodcont.2015.06.003

[57] Wang Y Wang L Xue H Qu W. A review of the growth of the fast food industry in China and its potential impact on obesity. Int J Environ Res Public Health. (2016) 13:1112. doi: 10.3390/ijerph13111112, PMID: 27834887 PMC5129322

[58] Rydell SA Harnack LJ Oakes JM Story M Jeffery RW French SA. Why eat at fast-food restaurants: reported reasons among frequent consumers. J Am Diet Assoc. (2008) 108:2066–70. doi: 10.1016/j.jada.2008.09.008, PMID: 19027410

[59] Husby I Heitmann BL Jensen KO. Meals and snacks from the child’s perspective: the contribution of qualitative methods to the development of dietary interventions. Public Health Nutr. (2009) 12:739–47. doi: 10.1017/S1368980008003248, PMID: 18671890

[60] Rosenheck R. Fast food consumption and increased caloric intake: a systematic review of a trajectory towards weight gain and obesity risk. Obes Rev. (2008) 9:535–47. doi: 10.1111/j.1467-789X.2008.00477.x, PMID: 18346099

[61] Dolton PJ Tafesse W. Childhood obesity, is fast food exposure a factor? Econ Hum Biol. (2022) 46:101153. doi: 10.1016/j.ehb.2022.101153 35809404

[62] Duncan JS Schofield G Duncan EK Rush EC. Risk factors for excess body fatness in New Zealand children. ASIA Pac J Clin Nutr. (2008) 17:138–47. PMID: 18364339

[63] Zhao Y Wang L Xue H Wang H Wang Y. Fast food consumption and its associations with obesity and hypertension among children: results from the baseline data of the childhood obesity study in China mega-cities. BMC Public Health. (2017) 17:933. doi: 10.1186/s12889-017-4952-x, PMID: 29212483 PMC5719642

[64] Farage MA Osborn TW MacLean AB. Cognitive, sensory, and emotional changes associated with the menstrual cycle: a review. Arch Gynecol Obstet. (2008) 278:299–307. doi: 10.1007/s00404-008-0708-2 18592262

[65] Chen Y Fang Z Zhu L He L Liu H Zhou C. The association of eating behaviors with blood pressure levels in college students: a cross-sectional study. Ann Transl Med. (2021) 9:155–5. doi: 10.21037/atm-20-8031, PMID: 33569457 PMC7867881

[66] Sadler JR Thapaliya G Jansen E Aghababian AH Smith KR Carnell S. COVID-19 stress and food intake: protective and risk factors for stress-related palatable food intake in U.S. adults. Nutrients. (2021) 13:901. doi: 10.3390/nu13030901, PMID: 33802066 PMC8000206

[67] Powell LM Han E Chaloupka FJ. Economic contextual factors, food consumption, and obesity among U.S. adolescents. J Nutr. (2010) 140:1175–80. doi: 10.3945/jn.109.111526 20392882

[68] Bohara SS Thapa K Bhatt LD Dhami SS Wagle S. Determinants of junk food consumption among adolescents in Pokhara Valley, Nepal. Front Nutr. (2021) 8:644650. doi: 10.3389/fnut.2021.644650, PMID: 33898498 PMC8060464

[69] Potvin Kent M Pauzé E Roy E-A de Billy N Czoli C. Children and adolescents’ exposure to food and beverage marketing in social media apps. Pediatr Obes. (2019) 14:e12508. doi: 10.1111/ijpo.12508 30690924 PMC6590224

[70] Government of Canada SC. The local restaurant environment in relation to eating out and sugary drink intake among Canadian children and youth. (2023). Available at: https://www150.statcan.gc.ca/n1/pub/82-003-x/2023008/article/00001-eng.htm (accessed October 28, 2024). 10.25318/82-003-x202300800001-eng 37647458

[71] Fleary SA Joseph P Chang H. Applying the information-motivation-behavioral skills model to explain adolescents’ fruits and vegetables consumption. Appetite. (2020) 147:104546. doi: 10.1016/j.appet.2019.104546, PMID: 31809812 PMC6957757

[72] Granner ML Sargent RG Calderon KS Hussey JR Evans AE Watkins KW. Factors of fruit and vegetable intake by Race, gender, and age among young adolescents. J Nutr Educ Behav. (2004) 36:173–80. doi: 10.1016/S1499-4046(06)60231-5 15544725

[73] Yang Y Dong B Zou Z Wang S Dong Y Wang Z. Association between vegetable consumption and blood pressure, stratified by BMI, among Chinese adolescents aged 13–17 years: a National Cross-Sectional Study. Nutrients. (2018) 10:451. doi: 10.3390/nu10040451 29621144 PMC5946236

[74] Shay CM Gooding HS Murillo R Foraker R. Understanding and improving cardiovascular health: An update on the American Heart Association’s concept of cardiovascular health. Prog Cardiovasc Dis. (2015) 58:41–9. doi: 10.1016/j.pcad.2015.05.003 25958016

[75] Collese TS Nascimento-Ferreira MV de Moraes ACF Rendo-Urteaga T Bel-Serrat S Moreno LA. Role of fruits and vegetables in adolescent cardiovascular health: a systematic review. Nutr Rev. (2017) 75:339–49. doi: 10.1093/nutrit/nux002, PMID: 28475799

[76] Hosseinpour-Niazi S Bakhshi B Betru E Mirmiran P Darand M Azizi F. Prospective study of total and various types of vegetables and the risk of metabolic syndrome among children and adolescents. World J Diabetes. (2019) 10:362–75. doi: 10.4239/wjd.v10.i6.362, PMID: 31231459 PMC6571485

[77] Martínez-Martínez MI Alegre-Martínez A Cauli O. Omega-3 long-chain polyunsaturated fatty acids intake in children: the role of family-related social determinants. Nutrients. (2020) 12:3455. doi: 10.3390/nu12113455, PMID: 33187190 PMC7697719

[78] Cave C Hein N Smith LM Anderson-Berry A Richter CK Bisselou KS. Omega-3 long-chain polyunsaturated fatty acids intake by ethnicity, income, and education level in the United States: NHANES 2003–2014. Nutrients. (2020) 12:2045. doi: 10.3390/nu12072045, PMID: 32660046 PMC7400855

[79] Noaghiul S Hibbeln JR. Cross-National Comparisons of seafood consumption and rates of bipolar disorders. Am J Psychiatry. (2003) 160:2222–7. doi: 10.1176/appi.ajp.160.12.2222, PMID: 14638594

[80] Lopata AL Lehrer SB. New insights into seafood allergy. Curr Opin Allergy Clin Immunol. (2009) 9:270–7. doi: 10.1097/ACI.0b013e32832b3e6f 19398906

[81] Qiu C Hou M. Association between food preferences, eating behaviors and socio-demographic factors, physical activity among children and adolescents: a cross-sectional study. Nutrients. (2020) 12:640. doi: 10.3390/nu12030640, PMID: 32121152 PMC7146169

[82] Liang K Chen S Chi X. Care their diet and mind: association between eating habits and mental health in Chinese left-behind children. Nutrients. (2022) 14:524. doi: 10.3390/nu14030524, PMID: 35276882 PMC8840046

[83] Jonsson KR Bailey CK Corell M Löfstedt P Adjei NK. Associations between dietary behaviours and the mental and physical well-being of Swedish adolescents. Child Adolesc Psychiatry Ment Health. (2024) 18:43. doi: 10.1186/s13034-024-00733-z, PMID: 38555430 PMC10981827

[84] Hoare E Hockey M Ruusunen A Jacka FN. Does fruit and vegetable consumption during adolescence predict adult depression? A longitudinal study of US adolescents. Front Psychiatry. (2018) 9:581. doi: 10.3389/fpsyt.2018.00581, PMID: 30483164 PMC6243081

[85] Park S Rim SJ Lee JH. Associations between dietary behaviours and perceived physical and mental health status among Korean adolescents. Nutr Diet. (2018) 75:488–93. doi: 10.1111/1747-0080.12444, PMID: 29978549

[86] An R Nickols-Richardson S Alston R Shen S Clarke C. Total, fresh, lean, and fresh lean beef consumption in relation to nutrient intakes and diet quality among U.S. adults, 2005–2016. Nutrients. (2019) 11:563. doi: 10.3390/nu11030563 30845714 PMC6471038

[87] Wiafe MA Apprey C Annan RA. Knowledge and practices of dietary iron and anemia among early adolescents in a rural district in Ghana. Food Sci Nutr. (2021) 9:2915–24. doi: 10.1002/fsn3.2249, PMID: 34136159 PMC8194733

[88] Murphy KJ Thomson RL Coates AM Buckley JD Howe PRC. Effects of eating fresh lean pork on Cardiometabolic health parameters. Nutrients. (2012) 4:711–23. doi: 10.3390/nu4070711, PMID: 22852059 PMC3407990

[89] Wall CR Stewart AW Hancox RJ Murphy R Braithwaite I Beasley R. The ISAAC phase three study group. Association between frequency of consumption of fruit, vegetables, nuts and pulses and BMI: analyses of the international study of asthma and allergies in childhood (ISAAC). Nutrients. (2018) 10:316. doi: 10.3390/nu10030316 29518923 PMC5872734

[90] Papanikolaou Y Slavin J Fulgoni VL. Adult dietary patterns with increased bean consumption are associated with greater overall shortfall nutrient intakes, lower added sugar, improved weight-related outcomes and better diet quality. Nutr J. (2024) 23:36. doi: 10.1186/s12937-024-00937-1, PMID: 38504300 PMC10953200

[91] Fernandes Gomes AP da Costa ACC Massae Yokoo E De MFV. Impact of bean consumption on nutritional outcomes amongst adolescents. Nutrients. (2020) 12:1083. doi: 10.3390/nu12041083, PMID: 32295142 PMC7230442

[92] Zou Y Huang L Zhao D He M Han D Su D. Food and nutrient intake in children and adolescents with or without overweight/obesity. Nutrients. (2023) 15:4450. doi: 10.3390/nu15204450, PMID: 37892525 PMC10609921

[93] Wang Z Zhang B Zhai F Wang H Zhang J Du W. Fatty and lean red meat consumption in China: differential association with Chinese abdominal obesity. Nutr Metab Cardiovasc Dis. (2014) 24:869–76. doi: 10.1016/j.numecd.2014.03.002, PMID: 24795160 PMC4112159

[94] Svisco E Byker Shanks C Ahmed S Bark K. Variation of adolescent snack food choices and preferences along a continuum of processing levels: the case of apples. Food Secur. (2019) 8:50. doi: 10.3390/foods8020050, PMID: 30717139 PMC6406983

[95] Wang Y Bentley ME Zhai F Popkin BM. Tracking of dietary intake patterns of Chinese from childhood to adolescence over a Six-year follow-up period. J Nutr. (2002) 132:430–8. doi: 10.1093/jn/132.3.430, PMID: 11880567

[96] World Health Organization. (2024) Healthy diet. Available at: https://www.who.int/news-room/fact-sheets/detail/healthy-diet (accessed august 12, 2024).

[97] Arcan C Neumark-Sztainer D Hannan P van den Berg P Story M Larson N. Parental eating behaviours, home food environment and adolescent intakes of fruits, vegetables and dairy foods: longitudinal findings from project EAT. Public Health Nutr. (2007) 10:1257–65. doi: 10.1017/S1368980007687151, PMID: 17391551

[98] Grosso G Marventano S Nolfo F Rametta S Bandini L Ferranti R. Personal eating, lifestyle, and family-related behaviors correlate with fruit and vegetable consumption in adolescents living in Sicily. IJVNR. (2014) 83:355–66. doi: 10.1024/0300-9831/a000177, PMID: 25497779

